# Editorial: Economic growth and health expenditures relationship between OECD countries

**DOI:** 10.3389/fpubh.2023.1322388

**Published:** 2023-11-07

**Authors:** Dilaver Tengilimoğlu

**Affiliations:** Department of Business, School of Business, Atilim University, Ankara, Türkiye

**Keywords:** health economics, public health, health services, health management, public health policy, health expenditures, economic growth, OECD

Countries need to increase the welfare and the wellbeing of their population. Countries' health systems are naturally essential in sustaining and increasing welfare and wellbeing. Furthermore, health systems are financed through health expenditures. Therefore, increasing health expenditures may positively impact the performance of health systems. Thus, increasing health expenditures in a country may increase the population's welfare and wellbeing.

A healthy population has many benefits, and potentially increasing economic growth is one of its most important benefits. Economic growth is vital for countries, so the relationship between economic growth and health expenditure must be carefully analyzed. Furthermore, the Organization for Economic Cooperation and Development (OECD) is one of the most prominent organizations in the world. In this context, this Research Topic focused on the relationship between economic growth and health expenditures among OECD countries. There were valuable contributions to the Research Topic, and nine articles were chosen.

Celik et al. analyze the relationship between health expenditures and economic growth with the convergence hypothesis and non-linear unit root tests in their contributed article titled “*Convergence of economic growth and health expenditures in OECD countries: evidence from non-linear unit root tests*”. They use data from 22 OECD countries from 1976 to 2020 and find that health expenditure convergence had significantly contributed to growth convergence. Beylik et al. analyze the same relationship with the Driscoll-Kraay standard error approach in their contributed article titled “*The relationship between health expenditure indicators and economic growth in OECD countries: a Driscoll-Kraay approach*”. They use data from 21 OECD countries and find that increasing health expenditures may increase economic growth.

Sustainability must accompany economic growth because countries cannot keep increasing their economic growth if it harms the population's health and the planet. Manzoor et al. find that innovation is critical for economic stability in their contributed article titled “*Sustainability-oriented innovation system and economic stability of the innovative countries*”, and they emphasize the importance of sustainability-oriented innovation. Their study obtains data from the 12 most innovative countries from 2011 to 2021 and uses fixed-effect methods.

Health outcomes are considered among the important indicators of the welfare and wellbeing of the population. Anwar et al. use the system generalized method of moments (GMM) in their contributed article titled “*Government health expenditures and health outcome nexus: a study on OECD countries*” and analyze the impact of health expenditures on health outcomes in the OECD countries. They find that health expenditures have a positive impact on life expectancy and a negative impact on infant mortality.

Treatable mortalities are very significant because they highlight the importance of health expenditures and economic growth. Countries with higher health expenditure and economic growth are more likely to have better and more effective health systems than countries with lower health expenditure and economic growth. As a better and more effective health system has a higher chance of treating treatable mortalities, higher health expenditure and economic growth can lower treatable mortality rates. Ivankova et al. investigate the relationships between health spending, treatable respiratory mortality, and GDP in OECD countries in their contributed article titled “*Understanding the relationships between health spending, treatable mortality and economic productivity in OECD countries*”. Their data covers the 1994–2016 period, and they find a negative relationship between health spending and treatable respiratory mortality in their regression analysis.

Food insecurity is another major issue that needs to be considered, as a healthy population depends on nutrients. Yılmaz and Günal evaluate food insecurity risk among 14 OECD countries in their contributed article titled “*Food insecurity indicators of 14 OECD countries in a health economics aspect: a comparative analysis*”. They find that food insecurity could be reduced by promoting economic growth.

Global pandemics such as the COVID-19 pandemic create a public health security issue that challenges health systems and disrupts the global economy. Fu et al. analyze the spatial linkage mechanism of public health security, better health status, and economic climate in their contributed article titled “*The spatial linkage mechanism: medical level, public health security, and economic climate from 19 OECD EU countries*”. They use data from 19 OECD European Union countries and find that increasing the medical level may reduce the negative impact of public health security issues on the economy.

Increasing health expenditures is beneficial, but only if it is effective. Therefore, analyzing the effectiveness of health expenditures is very important and necessary. The effectiveness of health expenditures becomes an even more significant concern during pandemics. Boduroglu et al. examine the effectiveness of health expenditures in 5 OECD countries during the COVID-19 pandemic in their contributed article titled “*Phase and wave dependent analysis of health expenditure efficiency: a sample of OECD evidence*”. They conduct unit root tests and find that the countries started to control the number of COVID-19 cases in the relaxation phase and at the beginning of the second wave of the COVID-19 pandemic by taking adequate measures, thereby increasing the effectiveness of health expenditures.

Furthermore, while increasing health expenditures is important and beneficial, it is also fundamental because it can financially ruin poor households, which is a big problem for the entire health system, especially in the long run. This is called catastrophic health expenditure. Söyük conducts a regression analysis with data from OECD countries for the 2003–2019 period and analyzes the impact of the pre-paid financing model implementations, such as those based on taxation, on the risk of catastrophic health expenditure in their contributed article titled “*The impact of public health expenditure and gross domestic product per capita on the risk of catastrophic health expenditures for OECD countries*”. They find that pre-paid financing models can reduce the risk of catastrophic health expenditure.

It is hoped that this Research Topic and these highlights stimulate further research about the relationship between economic growth and health expenditures.

## Author contributions

DT: Writing—original draft, Writing—review & editing.

